# Post-partum weight retention in Northeastern Brazilian women: a prospective NISAMI cohort study

**DOI:** 10.1590/1516-3180.2023.0084.R1.010623

**Published:** 2024-04-08

**Authors:** Sheila Monteiro Brito, Jerusa da Mota Santana, Marcos Pereira, Djanilson Barbosa Santos, Ana Marlucia Oliveira

**Affiliations:** I MSc, PhD. Adjunct Professor, Health Sciences Center, Universidade Federal do Recôncavo da Bahia (UFRB), Santo Antônio de Jesus (BA), Brazil.; II MSc, PhD. Adjunct Professor, Health Sciences Center, Universidade Federal do Recôncavo da Bahia (UFRB), Santo Antônio de Jesus (BA), Brazil.; III MSc, PhD. Adjunct Professor, Instituto de Saúde Coletiva (ISC), Universidade Federal da Bahia (UFBA), Salvador (BA), Brazil.; IV MSc, PhD. Adjunct Professor, Health Sciences Center, Universidade Federal do Recôncavo da Bahia (UFRB), Santo Antônio de Jesus (BA), Brazil.; V MSc, PhD. Full Professor, School of Nutrition, Universidade Federal da Bahia (UFBA), Salvador (BA), Brazil.

**Keywords:** Body weight changes, Pregnancy, Weight gain, Risk factors, Weight changes, body, Dietary patterns, Postpartum weight retention

## Abstract

**BACKGROUND:**

Weight retention during the post-partum period is associated with excessive weight gain.

**OBJECTIVES:**

To investigate factors associated with maternal weight retention at six months post-partum (PPWR).

**DESIGN AND SETTING:**

A prospective cohort study was conducted with 127 women monitored using prenatal services.

**METHODS:**

The outcome variable was represented by post-partum maternal weight retention and calculated as the difference between the mother’s weight at sixth month post-partum and her pregestational weight.

**RESULTS:**

The mean age of the pregnant women was 26.7 ± 5.25 years old, and the post-partum maternal weight retention was 46.5%. The proximal determinants showed a direct association with PPWR after adjusting for the distal and intermediate variables: excessive gestational weight gain (odds ratio [OR]:3.34; confidence interval [CI]:1.16–9.59), greater adhesion to dietary intake pattern 2 (composed of red meats and derivatives, eggs, industrialized foods, and coffee) (OR:2.70; CI:1.16–6.32), and the absence of exclusive maternal breastfeeding in the first month (OR:3.40; CI:1.27–9.12), as well as primiparity (OR:2.36; CI:1.00–5.55), an intermediate determinant. Insufficient weight gain in pregnancy was inversely associated with the outcome (OR:0.35; CI:0.31–0.93).

**CONCLUSIONS:**

Among the hierarchical determinants, proximal factors were interrelated with maternal weight retention, indicating that excessive total weight gain, an inadequate dietary intake pattern, and the absence of exclusive maternal breastfeeding in the first month of life work as dampeners of the return to pre-gestational weight. Prepartum and post-partum care interventions can contribute to reducing excess weight in women.

## INTRODUCTION

Nutritional alterations in the pregnancy-puerperal cycle, expressed as weight accumulation during pregnancy and the post-partum period, represent a risk factor for maternal weight retention. In a relatively short period, the modifications that occur in this stage of life are characterized by an expressive increase in nutrient demands, a high dietary intake, excessive weight gain, and lifestyle changes, which can represent etiological factors of weight retention in women.^1^ Research regarding post-partum maternal weight retention is in its infancy, both in Brazil and globally. A systematic review including post-partum studies from various countries indicates that 14 to 20% of women present > 5 kg of mean body weight retention from the sixth to the eighteenth month post-partum, varying from 0.5 to 4.0 kg.^2^ In Brazil, a review which included studies between 1997 and 2008 recorded a 14 to 65% variation in PPWR that was above expectations and a significant association with excessive gestational weight gain, indicating population differences.^3^ However, the risk and impact of weight accumulation on the body fat composition of women in the long run still need investigation.

Studies have shown relationship between post-partum weight gain and factors represented by an inadequate maternal diet in the pre- and peri-conceptional periods through pre-gestational excess weight or obesity, physical inactivity during pregnancy, unfavorable socioeconomic and demographic factors, multiparity, insufficient maternal breastfeeding time, or an inadequate type of dietary regime for the breastfed child in the first six months of life.^4-7^

These factors often intract with the different determinants of the hierarchy (distal, intermediate, and proximal) situated in other spheres of society and the environment in which the woman and her child live, configuring the health-disease phenomenon in this life stage. We identified a multifaceted relationship between post-partum maternal weight retention and various factors associated with this outcome. Thus, a hierarchical approach is indicated as a methodology for covering the complexity of this association, considering the different life contexts of the mother and child. However, not much information is generated based on the hierarchical approach to health-disease phenomena using selected observations in the pregnancy-puerperal cycle.

Further studies with different methodologies and results are required to draw consistent evidence for population groups living in diverse geographical regions and under different living conditions. Thus, this study aimed to provide insights into the factors associated with weight retention at the end of the sixth post-partum month.

## OBJECTIVE

This study aimed to analyze the factors associated with maternal weight gain in the sixth-month post-partum period.

## METHODS

### Study design and sample

In this prospective cohort, pregnant women were monitored in a municipality in the northeaster regionof Brazil from April 2012 to August 2014 as part of the prenatal service provided by the Health Units.

**Figure 1.**
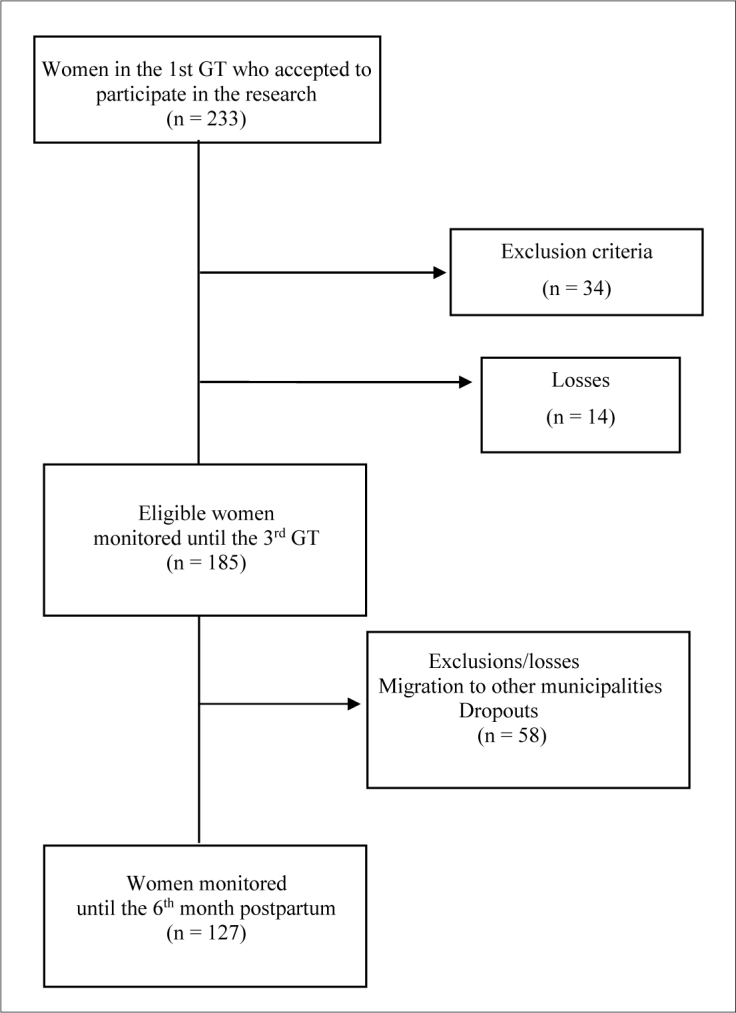
Cohort flowchart for capturing the sample, Santo Antônio de Jesus, Bahia, 2012-2014. GT = gestational trimester.

A total of 233 pregnant women suitable for participation in prenatal services were identified. After applying the inclusion criteria, 185 healthy pregnant women residing in an urban area of the municipality, aged 18 years or older, had a single pregnancy of up to 14 weeks at the time of eligibility, proved by ultrasound, were free of previous diseases or pregnancy-related complications, and had completed gestational follow-up were included. Of these, 58 either migrated to other municipalities or dropped out of participation in the post-partum stage. Thus, the participation of 127 women was recorded, for whom almost all the necessary information for the second stage of the study was available (Figure 1).

The duration of follow-up for the pregnant women was 12 months, with six months at each stage, the first corresponding to the pregnancy phase and the second to the post-partum phase.

Considering that the sample was not estimated to analyze maternal weight gain and associated factors, the power to detect and identify excessive maternal weight gain was calculated a posteriori. Under these circumstances, the calculated power was 99%, with a 5% margin of error and a 95% confidence interval (CI).

### Data collection and measurements

At the beginning of the study, pregnant women were enrolled in the study at Family Health Units. The following data were collected.

**Sociodemographic and lifestyle data:** During the prenatal visit, the women provided information on the socioeconomic and demographic conditions of their family, lifestyle habits, and prenatal care, which were recorded in a structured questionnaire.

**Reproductive and obstetric conditions:** Gestational age was calculated either based on the last menstrual cycle date, available on the pregnant woman’s chart, or the gestational age as recorded from the first ultrasound performed at the end of the first trimester.

Anthropometric data: The pre-gestational body mass index (pgBMI) was used to evaluate the pre-gestational maternal anthropometric status. This index was obtained as the ratio between pre-gestational weight ( kg) and height squared (m^2^), classified based on the parameters of the Institute of Medicine (IOM).^8^ Pre-gestational weight (PGW) was collected from the Pregnant Woman’s card.

**Food and dietary data:** Dietary intake during pregnancy was investigated at the first visit (8–14 weeks of gestation) using a semi-quantitative food frequency questionnaire adapted and prevalidated for the study population, composed of 73 food items. Details of the food consumption assessment have been recorded in a previous study.^9^

The woman’s dietary intake was assessed using the factor analysis technique with the extraction of principal components, considering a factor loading ≥ 0.4 for the composition of each pattern 9. The dietary groups were aggregated into four patterns: pattern 1 (cereals, roots, and tubers group; vegetables and legumes group; white meats); pattern 2 (red meat and egg group; meat products [in Portuguese: carne do sol, carne de sertão] and sausages group; industrialized foods group; coffee); pattern 3 (legumes group; fruits group; milk and dairy products group); and pattern 4 (sugars and sweet group; fats and fried snacks group).

### Pregnancy follow-up

Weight gain during pregnancy was used to assess and monitor the adequacy of weight gain during the gestational period. Anthropometric measurements were collected in the first, second, and third trimesters and were calculated in duplicate by a nutritionist and duly trained students from the health sector following standardized procedures.^10^ A maximum variation of 0.5 cm and 100 g was accepted for the height and weight measurements, respectively. A portable digital balance was used to measure weight (Marte, São Paulo, SP, Brazil) with a 150 kg capacity and 100 g sensitivity was previously calibrated and reassessed periodically. The mother’s height was calculated using a portable stadiometer with a capacity of 2000 mm and 0.5 cm sensitivity (Welmy S.A. São Paulo, SP, Brazil).

Variation in weight gain was used to evaluate weight increase during pregnancy. It was calculated based on the difference between the mother’s weight at the end of pregnancy and her pre-gestational weight.

The classification of the increase in weight gain followed the IOM recommendations,^8^ in which an increase of 12.5 to 18.0 kg was considered adequate for women who began their pregnancy with a low weight (pgBMI < 18.5 kg/m^2^), 11.5 to 16.0 kg for eutrophic women (pgBMI = 18.5 to 24.9 kg/m^2^), 7.0 to 11.5 kg for overweight women (pgBMI = 25.0 to 29.9 kg/m^2^), and 5.0 to 9.0 kg for obese women at the start of the pregnancy (pgBMI > 30.0 kg/m^2^). We considered the gestational weight increase excessive when it exceeded the limits recommended by the IOM for each pg BMI range and insufficient when it fell below the recommended level.^8^

In the post-partum phase, the women’s homes were visited twice. The mothers provided information about the delivery and health conditions of both themselves and their newborns, recorded using a standardized questionnaire.

**Breastfeeding:** The dietary intake of breastfed children was investigated, focusing on their dietary regime and the age at which each food or dietary group was first introduced.

**Post-partum maternal weight retention** During the post-partum period, anthropometric measurements at birth were collected by a trained technician from the maternity unit using calibrated equipment provided by the research team and recorded in a health booklet for children. Additional information regarding birth weight was obtained from the Live Birth Information System.

The cut-off point proposed by Ruesten et al. was considered to classify the intensity of PPWR.^1^ Thus, ≥ 5% pre-gestational weight retention was classified in the risk category^1^ as reference category (0), and < 5% pre-gestational weight retention was considered.

### Hierarchical approach

The outcome variable of this study was represented by post-partum maternal weight retention, which was calculated as the difference between the mother’s weight in the sixth month post-partum and her pregestational weight.

**Figure 2.**
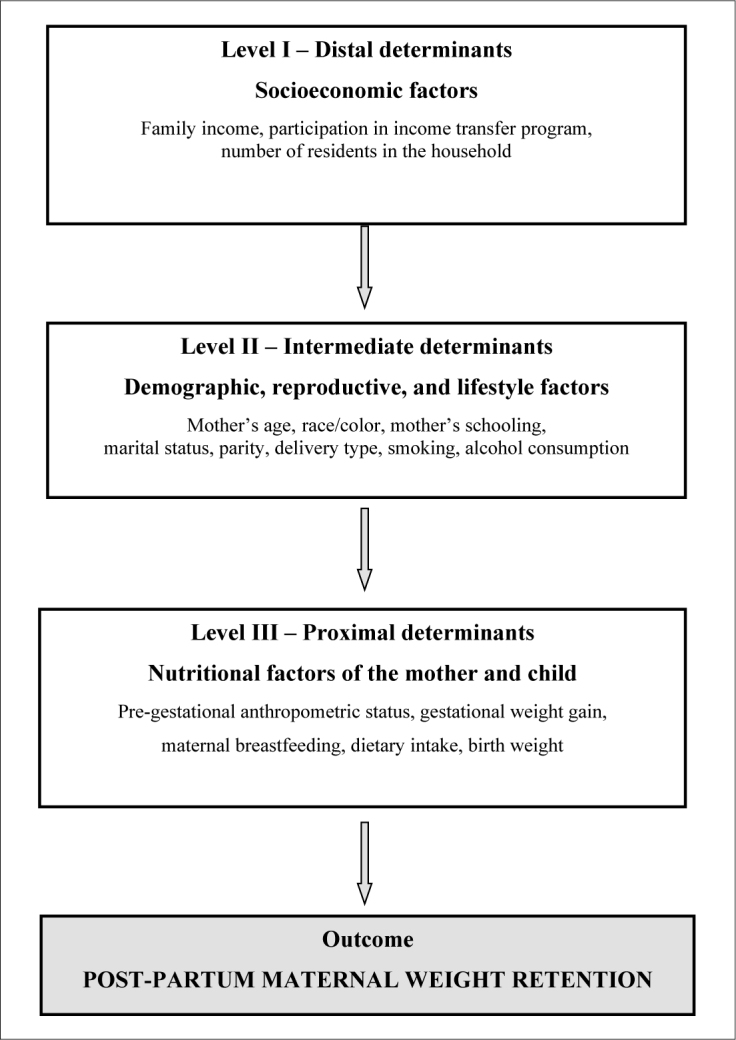
Hierarchical structure of the analysis of the factors associated with post-partum maternal weight retention, Santo Antônio de Jesus, Bahia, 2012-2014.

The exposure variables were included in the statistical analysis model of this study according to hierarchical levels (Figure 2). Thus, the following socioeconomic factors were considered at the distal level of determination: family income per capita (0 = 1/2 MW; 1 = ≤ 1/2 MW), participation in an income-based transfer program (0 = no; 1 = yes), and the number of residents in the household (0 = up to 4; 1 = more than 4). At the intermediate level of determination, the following sociodemographic, reproductive, and lifestyle factors were included: the mother’s age (0 = ≤ 21 years old; 1 = > 21 years old), the mother’s schooling (0 = > 8 years of study; 1 = ≤ 8 years of study), self-reported race/colour (0 = others; 1 = black), marital status (0 = with a partner; 1 = no partner), parity (0 = 1 child or more; 1 = primiparous), type of delivery (0 = natural; 1 = cesarean), alcohol consumption (0 = no; 1 = yes), and smoking (0 = no; 1 = yes).

At the proximal level of determination, the following maternal nutritional and lifestyle conditions were included: pre-gestational anthropometric nutritional status (0 = adequate; 1 = inadequate), weight gain during pregnancy (0 = low or adequate; 1 = excessive), dietary intake in pregnancy assessed according to the dietary patterns (0 = above the median; 1 = below the median), exclusive provision of maternal milk in the first month (0 = yes; 1 = no), exclusive provision of maternal milk in the sixth month (0 = yes; 1 = no), and weight of the newborn (0 = adequate ≥ 3000 g; 1 = insufficient < 3000 g).

### Statistical analysis

We used a logistic regression technique with a hierarchical approach to analyze the association between exposure and response variables. PPWR was used as the dependent variable. The exposure variables were allocated at the levels of determination (level I or distal, involves socioeconomic factors, level II or intermediate, is represented by maternal sociodemographic, reproductive, and lifestyle factors, and level III or proximal, covers the nutritional conditions of the woman and child) (Figure 2).

Initially, the consistency of the data was evaluated. Descriptive statistics were used to estimate the occurrence measures of all independent variables using the chi-square test or Fischer’s exact test at a 5% significance level.

We used the backward technique to select the variables that should compose the model, adopting the criterion of P < 0.20 with statistical significance in the bivariate analysis. Thus, all the exposure variables whose relationship with the response variable was <0.20 formed part of the multivariate model.

In the first stage of the multivariate analysis, we included all level I or distal (socioeconomic) factors, progressively eliminating them until only those whose association with post-partum weight retention (PPWR) generated a P value < 0.05 remained. In the second phase of the analysis, we included level II variables of the intermediate determinants (sociodemographic, reproductive, and lifestyle factors) adjusted by level I variables. Level II variables were then chosen, and those that had a significant statistical association were maintained in the model. The same procedure was employed to test the association between the third hierarchical level (proximal) variables (nutritional conditions of the mother and child) and the event, adjusted for Level I and II variables. All statistically significant (P < 0.05) associations formed part of the final model.

Excel software was used to input the dietary intake data, SSPSS (version 17.0; SPSS, Chicago, United States) for data entry and factor analysis, and STATA 10.0 (Stata Corporation, College Station, Texas, United States) for multivariate modeling.

### Ethical approval

The Faculdade Adventista da Bahia’s Ethics Committee for Research Involving Human Beings granted approval (No. 4369.0.000.070-10) for this study on September 14, 2010. All study procedures were performed in accordance with the Declaration of Helsinki, code of ethics established by the World Medical Association for human experiments. Informed consent was obtained for the experimentation with human subjects, and the privacy rights of human subjects were observed.

## RESULTS

### Description of participants

There were 127 women in this study, with a mean age of 26.7 ± 5.25 years old. The frequency of post-partum maternal weight retention was 46.5%, and the mean was 6.58 ± 5.98 kg. At the start of pregnancy, the mean pgBMI was 24.46 ± 4.92 kg/m^2^ and 28.57 ± 4.34 kg/m^2^ at the end. The mean gestational weight gain was 11.4 ± 9.20 kg, which was adequate in 22.8% of the cases. Their mean height was 1.59 ± 0.06 m. The total prevalence of maternal breastfeeding was 59.1% in the sixth month post-partum and was exclusive in 15% of cases. The mean age at which complementary foods were introduced into the breastfed children’s diet was 3.47 ± 2.37 months (data not presented).

### Main analysis

The results of the bivariate analysis are shown in Table 1. The family income per capita variable (P = 0.48) (level I), although it did not have a P value ≤ 0.20, was included in the multivariate analysis model due to the association of this variable with health and nutrition events, showing the pertinence of its inclusion in the statistical model because of its epidemiological relevance.

**Table 1.** Socioeconomic characteristics (distal determinants), sociodemographic, reproductive, and lifestyle characteristics (intermediate determinants), and nutritional characteristics of the woman and child (proximal determinants), according to post-partum weight retention, in Santo Antônio de Jesus, BA, 2012–14

Distal determinants	n	Weight retention^*^		P value
		Yes	No	
		n (%)	n (%)	
**Income per capita** ^**^				
≥ 1/2 MW	90	40 (44.44)	50 (55.56)	0.48
< 1/2 MW	37	19 (51.35)	18 (48.65)	
**Number of residents in the household**				
Up to 4 people	109	52 (47.71)	57 (52.29)	0.49
> 4 people	18	7 (38.39)	11 (61.11)	
**Participation in an income transfer program**				
No	103	49 (47.57)	54 (52.43)	0.60
Bolsa Família program	24	10 (41.67)	14 (58.33)	
**Intermediate Determinants**				
**Mother’s age**				
> 21 years old	103	44 (42.72)	59 (57.28)	0.08
≤ 21 years old	24	15 (62.50)	9 (37.50)	
**Race/color**				
Others^***^	74	38 (51.35)	36 (48.65)	0.19
Black	53	21 (39.62)	32 (60.38)	
**Mother’s schooling**				
> 8 years	87	42 (48.28)	45 (51.72)	0.54
≤ 8 years	40	17 (42.50)	23 (57.50)	
**Marital status**				
With a partner	116	55 (47.41)	61 (52.59)	0.48
No partner	11	4 (36.36)	7 (63.64)	
**Parity**				
Primiparous	67	36 (53.73)	31 (46.27)	0.08
≥ 1 child	60	23 (38.33)	37 (61.67)	
**Delivery type**				
Normal	29	18 (62.07)	11 (37.93)	0.06
Cesarean	98	41 (41.84)	57 (58.16)	
**Alcohol consumption**				
Yes	84	43 (51.19)	41 (48.81)	0.14
No	43	16 (37.21)	27 (62.79)	
**Smoking**				
Yes	22	12 (54.55)	10 (45.45)	0.40
No	105	47 (44.76)	58 (55.24)	
**Proximal determinants**				
**Pre-gestational body mass index**				
Adequate	70	34 (48.57)	36 (51.43)	0. 60
Inadequate	57	25 (43.86)	32 (56.14)	
**Weight gain during pregnancy**				
Insufficient	49	12 (24.49)	37 (75.51)	0.00
Adequate	43	22 (51.16)	21 (48.84)	
Excessive	35	25 (71.43)	10 (28.57)	
**Exclusive breastfeeding up tosixth month**				
Yes	19	5 (26.32)	14 (73.68)	0.06
No	108	54 (50.00)	54 (50.00)	
**Exclusive breastfeeding in the first month**				
Yes	96	40 (41.67)	56 (58.33)	0.06
No	31	19 (61.29)	12 (38.71)	
**Dietary intake (Pattern 1)** ^****^				
Adequate	68	31 (45.59)	37 (54.41)	0.83
Inadequate	59	28 (47.46)	31 (52.54)	
**Dietary intake (Pattern 2)** ^****^				
Adequate	64	28 (43.75)	36 (56.25)	0.04
Inadequate	63	39 (61.90)	24 (38.10)	
**Dietary intake (Pattern 3)** ^****^				
Adequate	72	33 (45.83)	39 (54.17)	0.87
Inadequate	55	26 (47.27)	29 (52.73)	
**Dietary intake (Pattern 4)** ^****^				
Adequate	64	30 (46.88)	34 (53.13)	0.92
Inadequate	63	29 (46.03)	34 (53.97)	
**Weight (newborn)**				
Adequate (≥ 3000 g)	100	47 (47.00)	53 (53.00)	0.81
Insufficient (< 3000 g)	27	12 (44.44)	15 (55.56)	

* Refers to the sixth month of the post-partum period;

** Baseline minimum wage = 622.00 BRL;

*** Race/color variable, the “others” category included white, brown, indigenous, and yellow;

**** Pattern 1 (cereals, roots, and tubers; vegetables and legumes; white meats); Pattern 2 (red meats and eggs; meat products and sausages; industrialized foods; coffee); Pattern 3 (legumes; fruits; milk and dairy products); and Pattern 4 (sugars and sweets; fats and fried snack).

Among the sociodemographic, reproductive, and lifestyle factors (level II – intermediate), the parity variable (odds ratio [OR]:2.36; CI:1.00–5.55) was identified after adjusting for the family income variable. The results of the multivariate analysis for the level III (proximal) variables, adjusted by the level I and II variables, indicated that excessive gestational weight gain (OR:3.34; CI:1.16–9.59), insufficient gestational weight gain (OR:0.35; CI:0.13–0.93), dietary intake pattern 2, composed of red meat and derivatives, eggs, sausages, industrialized foods, and coffee (OR:2.70; CI:1.16–6.32), and the absence of exclusive maternal breastfeeding in the first month (OR:3.40; CI:1.27–9.12) were associated with post-partum maternal weight retention in the final model (Table 2).

**Table 2.** Adjusted odds ratio of the determinant factors (distal, intermediate, and proximal) of weight retention in the sixth month post-partum period obtained through the hierarchized model of the logistic regression analysis, in Santo Antônio de Jesus, BA, 2012–14

Distal determinants: socioeconomic factors	OR	CI 95%	P value
**Income per capita**			
≥ 1/2 MW	1.00		
< 1/2 MW	2.05	0.79–5.29	0.138
Intermediate determinants: sociodemographic, reproductive, and lifestyle factors^*^			
**Race/color**			
Others	1.00	–	–
Black	0.59	0.25–1.37	0.220
**Parity**			
≥ 1 child	1.00	–	–
Primiparous	2.36	1.01–5.55	0.049
Proximal determinants: Nutritional characteristics of the mother and child^**^			
**Gestational weight gain**			
Adequate	1.00	–	–
Excessive	3.34	1.16–9.59	0.025
Insufficient	0.35	0.13–0.93	0.034
**Dietary intake (Pattern 2)**			
Adequate	1.00	–	–
Inadequate	2.70	1.16–6.32	0.022
**Exclusive breastfeeding in the first month**			
Yes	1.00	–	–
No	3.40	1.27–9.12	0.015

* The Level 2 association measures were adjusted by Level 1 variables;

** The Level 3 association measures were adjusted by Level 1 and 2 variables. OR = odds ratio; CI = confidence interval.

## DISCUSSION

The findings of this study indicate that women from a municipality in the northeastern region of Brazil had a high prevalence (46.5%) of maternal weight retention in the sixth post-partum month. We also observed factors associated with events at proximal and intermediate hierarchical levels. Thus, these results reaffirm the close relationship between hierarchical determinants and health conditions. The absence of exclusive maternal breastfeeding in the first month post-partum, greater adhesion to a dietary intake pattern based on red meat, eggs, industrialized products, processed foods, sausages, and coffee (proximal determinants), and primiparity (intermediate determinant) were associated with maternal weight retention. In addition, excessive gestational weight gain promoted PPWR, whereas insufficient gain dampened it (proximal determinant).

These results highlight that at six months post-partum, maternal weight retention > 5 kg is an important risk factor for long-term maintenance of excess weight, ^1,8^ reinforcing the hypothesis that this stage of life, is a period of risk for the occurrence of excess weight in the female population. In this study, women who gained excessive weight presented a 3.3 times higher risk (CI) for PPWR than those who showed weight gain within established limits.^8^

However, it was also observed that insufficient weight gain during pregnancy prevents weight retention, which would manifest itself in a reduction in the woman’s weight. Both situations are undesirable and may have a negative impact on women’s health because they can contribute to the accumulation or depletion of essential nutrients. Thus, in the first six months post-partum, the fat reserves accumulated during pregnancy should have already been mobilized for lactation, with energy and nutrient supplementation needed for breastfeeding continuity. In this sense, inadequate dietary intake and lifestyle habits lead to excess and insufficient weight gain in the pregnancy-puerperal cycle, resulting in risks to the health and nutrition of mother and child.

The recommendations^8^ are outlined considering the pre-gestational maternal anthropometric status and the results of monitoring weight gain during pregnancy. When dietary intake is above the level recommended for the body mass index range, reserves are also converted into body fat. However, in this situation, the diet exceeds the need for lactation expenditure and thus contributes to maternal weight accumulation. The results of this study are consistent with those of other studies conducted using different methodologies and populations, including those done in Brazil.^9,11^

The excessive consumption of red meats, meat products, sausages, industrialized foods, and coffee raises the risk of PPWR by 2.70 times (CI:1.16–6.32). It should be considered that although this pattern includes some foods that are sources of proteins with high biological value (meats and eggs) and iron (red meats), recommended for the gestational period, cultural factors also encourage these foods to be fried, adding unhealthy fats and increasing the calorie content and supply of sodium derived from salty meat and sausages, increasing the risk associated with them^9^ and corroborating other epidemiological studies on dietary intake during pregnancy.^11,12^

However, greater adhesion to healthy dietary patterns did not prevent PPWR in this study. The negative effect of the high total energy value of the diet may have a more significant dampening impact on the outcome, given the low maternal preference for healthier foods. Adhesion to healthy food patterns and their relationship with PPWR have been controversial in other investigations.^13^

Maternal breastfeeding, another proximal factor studied, generated results that support already existing evidences that it is a strong deterrent to adequate post-partum maternal weight.^7,14^ In this study, a greater risk of weight retention was observed in women who did not exclusively breastfeed during the first post-partum month. This period can be the most critical for mothers, as it implies lower energy expenditure for breastfeeding, consequently exacerbating weight accumulation, and also for the child because maternal milk may already be substituted for other foods not recommended during this stage of development.

Lactation implies a high mobilization of energy and nutrients derived from the diet and maternal reserves to meet nutritional demands. This is associated with greater energy expenditure and a greater decline in body weight accumulated during pregnancy. This finding is corroborated by studies that associate a longer exclusive breastfeeding time with a greater frequency of a return to pre-gestational weight.^14-18^ However, discrepantancies can be observed in some studies, resulting from the variations in aspects related to the regime, intensity, and duration of maternal breastfeeding. This may explain the controversial results regarding this subject.^19,20^

Primiparity was another reproductive factors at the intermediate determination level associated with weight retention in women.^7^ A study cohort of 12,875 women from Nova Scotia, Canada, showed that multiparous women who gained more weight than recommended by the GWG guidelines ended up retaining more post-partum weight (5.3 kg, 95% CI 5.1–5.5) than primiparous women ( 4.3 kg, 95% CI 4.0–4.7). ^21^However, the evidence for the role of parity in PPWR is inconclusive.^22^

This study found no significant association between socioeconomic and demographic variables and PPWR. We chose to maintain family income per capita (a distal level variable) to adjust the other model levels because of the known influence of unfavorable socioeconomic conditions and a precarious state of health and nutrition. Family income per capita may impact this stage of life by resulting in decresse access to prenatal care, less social and family support to care for the child, and greater barriers to weight control, which could favor weight accumulation in this phase.^23,24^

In this study, the factors influencing maternal weight retention were interrelated in determining the weight adequacy pattern in Brazilian women during the reproductive period. These results reinforce the epidemiological relevance of basic prenatal monitoring actions, including nutritional assessments focused on controlling weight gain, adequate dietary intake, and a healthy lifestyle, to contribute to the health care and nutrition of the infant-maternal group.

As limitations of the study, we identified a loss in the follow-up, a characteristic that represents an implicit challenge for longitudinal studies.^25^ The present investigation was conducted with a population in low socioeconomic conditions. Migration due to temporary (rented) residences, resulted in a 31.3% loss in the 12-month follow-up period. However, these losses were random for the studied variables, especially those associated with events.

The methodological care taken in this study and the appropriate statistical analyses used ensured reliable results compatible with those of the other studies mentioned here.

## CONCLUSION

The results of this study contribute to scientific knowledge on the determinant factors of PPWR in the context of the northeast region of Brazil, for which data on the occurrence and magnitude of the problem are scarce. Pre- and post-partum care interventions represent positive actions that could contribute to reducing excess weight in women. In light of these considerations, it is pertinent to recommend an increase in the number of studies on gestational weight gain and dietary intake during pregnancy and their relationship with PPWR, with larger samples and a population from different living conditions, to understand the complexity of the phenomena that determine this event.

## References

[1] von Ruesten A, Brantsæter AL, Haugen M (2014). Adherence of pregnant women to Nordic dietary guidelines in relation to post-partum weight retention: results from the Norwegian Mother and Child Cohort Study.. BMC Public Health..

[2] Gore SA, Brown DM, West DS (2003). The role of post-partum weight retention in obesity among women: a review of the evidence.. Ann Behav Med..

[3] Nogueira JL, Saunders C, Leal Mdo C (2015). Métodos antropométricos utilizados na avaliação da retenção do peso no período pós-parto: uma revisão sistemática [Anthropometric methods used in the evaluation of the postpartum weight retention: a systematic review].. Cien Saude Colet..

[4] Moore AP, Flynn AC, Adegboye ARA, Goff LM, Rivas CA (2021). Factors influencing pregnancy and post-partum weight management in women of African and Caribbean ancestry living in high income countries: systematic review and evidence synthesis using a behavioral change theoretical model.. Front Public Health..

[5] Zanotti J, Capp E, Wender MC (2015). Factors associated with post-partum weight retention in a Brazilian cohort.. Rev Bras Ginecol Obstet..

[6] Makama M, Skouteris H, Moran LJ, Lim S (2021). Reducing post-partum weight retention: a review of the implementation challenges of post-partum lifestyle interventions.. J Clin Med..

[7] Jiang M, Gao H, Vinyes-Pares G (2018). Association between breastfeeding duration and post-partum weight retention of lactating mothers: A meta-analysis of cohort studies.. Clin Nutr..

[8] Rasmussen KM, Yaktine AL, Institute of Medicine (US) and National Research Council (US) Committee to Reexamine IOM Pregnancy Weight Guidelines. (2009). Weight Gain During Pregnancy: Reexamining the Guidelines..

[9] Da Mota Santana J, Alves de Oliveira Queiroz V, Monteiro Brito S, Barbosa Dos Santos D, Marlucia Oliveira Assis A (2015). food consumption patterns during pregnancy: a longitudinal study in a region of the Northeast of Brazil.. Nutr Hosp..

[10] Lohman TG, Roche AF, Martorell R (1988). Anthropometric standardization reference manual..

[11] Coelho Nde L, Cunha DB, Esteves AP, Lacerda EM, Theme Filha MM (2015). Dietary patterns in pregnancy and birth weight.. Rev Saude Publica..

[12] Martins AP, Benicio MH (2011). Influence of dietary intake during gestation on post-partum weight retention.. Rev Saude Publica..

[13] Boghossian NS, Yeung EH, Lipsky LM, Poon AK, Albert PS (2013). Dietary patterns in association with post-partum weight retention.. Am J Clin Nutr..

[14] Tahir MJ, Haapala JL, Foster LP (2019). Association of full breastfeeding duration with post-partum weight retention in a cohort of predominantly breastfeeding women.. Nutrients..

[15] Brandhagen M, Lissner L, Brantsaeter AL (2014). Breastfeeding in relation to weight retention up to 36 months post-partum in the Norwegian Mother and Child Cohort Study: modification by socioeconomic status?. Public Health Nutr..

[16] Kac G, D’Aquino Benicio MH, Valente JG, Velásquez-Meléndez G (2003). Post-partum weight retention among women in Rio de Janeiro: a follow-up study.. Cad Saude Publica..

[17] Vinter CA, Jensen DM, Ovesen P (2014). Post-partum weight retention and breastfeeding among obese women from the randomized controlled Lifestyle in Pregnancy (LiP) trial.. Acta Obstet Gynecol Scand..

[18] Castillo H, Santos IS, Matijasevich A (2016). Maternal pre-pregnancy BMI, gestational weight gain and breastfeeding.. Eur J Clin Nutr..

[19] Coitinho DC, Sichieri R, D’Aquino Benício MH (2001). Obesity and weight change related to parity and breastfeeding among parous women in Brazil.. Public Health Nutr..

[20] Sichieri R, Field AE, Rich-Edwards J, Willett WC (2003). Prospective assessment of exclusive breastfeeding in relation to weight change in women.. Int J Obes Relat Metab Disord..

[21] Ashley-Martin J, Woolcott C (2014). Gestational weight gain and post-partum weight retention in a cohort of Nova Scotian women.. Matern Child Health J..

[22] Hill B, Bergmeier H, McPhie S (2017). Is parity a risk factor for excessive weight gain during pregnancy and post-partum weight retention? A systematic review and meta-analysis.. Obes Rev..

[23] Walker LO, Fowles ER, Sterling BS (2011). The distribution of weight-related risks among low-income women during the first post-partum year.. J Obstet Gynecol Neonatal Nurs..

[24] Monteiro da Silva M da C, Marlúcia Oliveira A, Pereira Magalhães de Oliveira L (2013). Determinants of post-partum weight variation in a cohort of adult women; a hierarchical approach.. Nutr Hosp..

[25] Rebelo F, Castro MBT, Dutra CL, Schlussel MM, Kac G (2010). Fatores associados à retenção de peso pós-parto em uma coorte de mulheres, 2005-2007.. Rev Bras Saude Mater Infant..

